# BI-2865, a pan-KRAS inhibitor, reverses the P-glycoprotein induced multidrug resistance in vitro and in vivo

**DOI:** 10.1186/s12964-024-01698-4

**Published:** 2024-06-13

**Authors:** Qihong Yang, Kenneth Kin Wah To, Guilin Hu, Kai Fu, Chuan Yang, Shuangli Zhu, Can Pan, Fang Wang, Kewang Luo, Liwu Fu

**Affiliations:** 1 People’s Hospital of Longhua, Shenzhen, 518109 China; 2 State Key Laboratory of Oncology in South China, Guangdong Provincial Clinical Research Center for Cancer, Sun Yat-sen University Cancer Center, Guangzhou, 510060 P. R. China; 3 School of Pharmacy, The Chinese University of Hong Kong, Hong Kong, 999077 China; 4 State Key Laboratory of Phytochemistry and Plant Resources in West China, Kunming Institute of Botany, Chinese Academy of Sciences, Kunming, 650201 China

**Keywords:** KRAS inhibitor, BI-2865, Multidrug resistance, ABC transporters, ABCB1/P-gp, Chemotherapeutic agent, Combination therapy

## Abstract

**Background:**

Multidrug resistance (MDR) limits successful cancer chemotherapy. P-glycoprotein (P-gp), BCRP and MRP1 are the key triggers of MDR. Unfortunately, no MDR modulator was approved by FDA to date. Here, we will investigate the effect of BI-2865, a pan-KRAS inhibitor, on reversing MDR induced by P-gp, BCRP and MRP1 in vitro and in vivo, and its reversal mechanisms will be explored.

**Methods:**

The cytotoxicity of BI-2865 and its MDR removal effect in vitro were tested by MTT assays, and the corresponding reversal function in vivo was assessed through the P-gp mediated KBv200 xenografts in mice. BI-2865 induced alterations of drug discharge and reservation in cells were estimated by experiments of Flow cytometry with fluorescent doxorubicin, and the chemo-drug accumulation in xenografts’ tumor were analyzed through LC-MS. Mechanisms of BI-2865 inhibiting P-gp substrate’s efflux were analyzed through the vanadate-sensitive ATPase assay, [^125^I]-IAAP-photolabeling assay and computer molecular docking. The effects of BI-2865 on P-gp expression and KRAS-downstream signaling were detected via Western blotting, Flow cytometry and/or qRT-PCR. Subcellular localization of P-gp was visualized by Immunofluorescence.

**Results:**

We found BI-2865 notably fortified response of P-gp-driven MDR cancer cells to the administration of chemo-drugs including paclitaxel, vincristine and doxorubicin, while such an effect was not observed in their parental sensitive cells and BCRP or MRP1-driven MDR cells. Importantly, the mice vivo combination study has verified that BI-2865 effectively improved the anti-tumor action of paclitaxel without toxic injury. In mechanism, BI-2865 prompted doxorubicin accumulating in carcinoma cells by directly blocking the efflux function of P-gp, which more specifically, was achieved by BI-2865 competitively binding to the drug-binding sites of P-gp. What’s more, at the effective MDR reversal concentrations, BI-2865 neither varied the expression and location of P-gp nor reduced its downstream AKT or ERK1/2 signaling activity.

**Conclusions:**

This study uncovered a new application of BI-2865 as a MDR modulator, which might be used to effectively, safely and specifically improve chemotherapeutic efficacy in the clinical P-gp mediated MDR refractory cancers.

**Supplementary Information:**

The online version contains supplementary material available at 10.1186/s12964-024-01698-4.

## Background

Although considerable progress has made in cancer therapy, chemotherapy is still the most important methods widely used in treating various malignant tumors. However, clinical studies showed that some cancer patients failed to respond or presented torpid to multiple chemotherapeutic drugs structurally and functionally different [1], which led out great attention on the tumor multidrug resistance (MDR) [2], a crucial obstacle to successful cancer chemotherapy. It is reported that more than 90% of mortality in cancer patients treated by conventional chemotherapeutics or targeted agents are due to multidrug resistance (MDR) [3]. MDR dramatically limited successful cancer chemotherapy.

MDR, also known as pleiotropic drug resistance, is a result of joint action of various factors and mechanisms involving increased permeability of cytomembrane, elevated drug efflux, enhanced DNA repair capacity, genetic alterations and so on [3–5]. Among these causes, high level of ATP-binding cassette (ABC) proteins on cell membrane of carcinomas is acknowledged as the most frequent event contributing to MDR, which is responsible for regulating the absorption, distribution and discharge of chemical compounds [6]. Highly conserved ABC transporters have a characteristic architecture composed of four domains: two nucleotide-binding domains (NBDs) in cytoplasm that couple ATP binding and hydrolysis, and two domains across cell membrane (TMDs) which recognize and transport substrates [7]. This family of ABC transporters is like pumps in cell membrane that actively control the intracellular amounts of substrates, its overexpression accounts for most phenomena of sub-therapeutic accumulation and lost efficacy of many chemotherapies [8–10]. Despite the significance of ABC transporters to cancer MDR has been well stated in vitro and in vivo, there are only limited agents like verapamil, valspodar, dofequidar, Ko143, MK571 have been developed as ABC transporter modulators [11, 12] but still with few success in clinical practice mainly for inefficiency, adverse side-effects, non-specificity and uncertain drug-interactions [13–15]. As a result, more promising vivo-effective agents to defeat MDR in cancer chemotherapy remain attractive and need to be further developed.

Among the many ABC transporters, subfamily members as P-glycoprotein (shorted as P-gp, encoded by *ABCB1* gene), BCRP (referred to the breast cancer resistance protein, encoded by *ABCG2* gene) and MRP1 (the multidrug resistance associated protein 1, encoded by *ABCC1* gene) are the key triggers of MDR [1, 16]. Numerous studies revealed a high correlation of these three ABC transporters with MDR cancers including leukemias, breast carcinomas, colon cancer and lung carcinomas in clinic, and their overexpression or hyperactivation notably portended a poor response to chemotherapy [17, 18], which makes them charming therapeutic targets to improve efficacy of chemo agents. Unfortunately, no MDR modulator was approved by FDA to date.

*KRAS* is one of the most common driven-genes studied extensively in human carcinogenesis, whose encoded protein KRAS elicits function of regulating intracellular signal cascades and diverse cellular processes as a GTPase transducer [19, 20]. Substantial documents show that KRAS activation mutations widely existed in sundry malignant carcinoma including pancreatic, colon cancers and lung cancers [21, 22], and closely involved with undesirable progression and prognosis in clinic cancer therapy [23, 24]. However, only the study of KRAS G12C mutation has made a breakthrough with a specific inhibitor AMG510 (Sotorasib) approved by FDA [25] and MRTX849 (Adagrasib) ongoing clinical trials [26], and the limited success just benefits to a very small number of lung cancer patients [27]. It is encouraging that a none-covalent pan-KRAS inhibitor, BI-2865, firstly published in Nature by Memorial Sloan-Kettering Cancer Center, exhibited excellent activity in blocking the great mass of KRAS variants including G12D/C/A/F/V/S, G13D/C, V14I, Q22K, L19F, D33E, K117N, Q61H, and A146T/V, and dramatically restricted KRAS-driven tumor growth without harming animal weight [28], suggesting BI-2865 with a good development and application prospect. Given the previous research on Adagrasib reversing MDR [29], herein, we will further explore the ability and mechanisms of BI-2865 to overcome ABC transporter-mediated MDR, which may provide new sources for developing new MDR modulators and produce more broad therapeutic implications in clinic oncology.

## Materials and methods

### Reagents

BI-2865 (C-1443) was purchased from the Chemgood (Henrico, VA, USA). Paclitaxel (HY-B0015), Verapamil (HY-14,275), Vincristine (HY-N0488), Doxorubicin (HY-15,142 A) and Topotecan (HY-13,768) were acquired from MedChem Express. Mitoxantrone (S2485), Cisplatin (S1166), Ko143 (S7043) and Thiazolyl Blue (MTT agents, S6821) were bought from Selleck. PEG300, Tween-80 and DMSO were from Sigma-Aldrich. Antibodies for P-gp (sc-13,131), MRP1 (sc-18,835) and GAPDH (AMM22048N) were bought from the Santa Cruz. BCRP antibody (#AB40537) was got from AbSci. Antibodies for p-AKT (80455-1), AKT (60203-2), p-ERK1/2 (28733-1-AP) and ERK1/2 (28733-1-AP) were obtained from Proteintech. High-glucose DMEM, RPMI-1640, penicillin-streptomycin solution and trypsin-EDTA were bought from Gibco BRL. The Fetal Bovine Serum (FSP500) was got from ExCell Bio.

EZ-press RNA Purification Kit (B0004D), 4× Reverse Transcription Mix (EZB-RT2GQ), 2× SYBR qPCR Mix (A0001-R2) were purchased from the ZScience Biotechnology Corporation Limited on biotechnology (EZBioscience, USA).

### Cell lines and culture

Both DMEM and RPMI-1640 were added with FBS (10%) and penicillin-streptomycin (1%) to mix for completed. Human oral cancer KB cell line and its P-gp overexpressed MDR KBv200 cell line, leukemia HL60 cell line and its MRP1-overexpressed MDR HL60/adr cell line were fostered with the completed RPMI-1640 medium. Human breast malignancy MCF-7 cell line and the corresponding P-gp overexpressed MCF-7/adr cell line, colon tumor S1 cell line and its BCRP-overexpressed S1-M1-80 cell line, and the human immortalized HEK293 cell lines stable-transfected with *ABCB1, ABCG2-*482-R2 or *ABCG2-*482-T7, and its corresponding HEK293/Vector cell line [30] were cultured with the completed DMEM medium. All mentioned cell lines were cultivated in an incubator of 37 ℃ temperature and 5% CO2.

### Cytotoxicity and MDR reversal assessed by MTT assays

Planted 2000–3000 cells into the 96-well plates. When cells got attached, treated them with desired concentration of BI-2865 for 72 h, then incubated cells (200 µL medium) with 20 µL MTT (5 mg/mL) regents at 37 ℃ for another 4 h, followed by dissolving the crystal with 150 µL DMSO. Finally, detecting the OD absorption at 570/630 nm.

To evaluate the MDR reversal effect of BI-2865, cancer cells were treated with indicated BI-2865, Verapamil, Ko143 or MK571 at first, subsequently added gradient-diluted desired chemo-drugs. After incubation for 72 h, detecting the cell viability by MTT as described above.

### Animal experiments

The ethical approval number (No. L102042023100C) of animal experiments in this study was authorized by Animal Ethics Committee of Sun Yat-sen University Cancer Center. All animal experiments were performed by strictly following the Declaration of Helsinki.

To assess the reversal action of BI-2865 in vivo, 3.5 × 10 ^6^ KBv200 cells resuspended in PBS were subcutaneously seeded to the 3-week female BALB/C nude mice. As the tumors grew to nearly 100 mm^3^, randomly grouping mice into four and giving indicated administrations once every two days: (a) Saline (as Control); (b) paclitaxel intraperitoneal injection (15 mg/kg); (c) BI-2865 gavage (30 mg/kg, dissolved in solution of 10% DMSO, 40% PEG300, 5% Tween-80 and 45% Saline); (d) Co-administration of BI-2865 and paclitaxel. Simultaneously, recorded mice body weight and the tumor length and width. The tumor volume (V) was assessed by (length × width ^2^ /2). All mice were subject to euthanasia when tumor average volume in the saline group reached about 2000 mm^3^, and tumors were excised and weighed.

### Doxorubicin accumulation and efflux assays

The excitation and emission wavelength of Doxorubicin (DOX) is correspondingly 475–485 nm and 575–585 nm. DOX amount in cells can be tested by Flow cytometry.

For analyzing the influence of BI-2865 on DOX accumulating in MDR cells, cells were pretreated by different doses of BI-2865 or vehicle for 3 h, followed by 10 µM Dox incubation for another 3 h. In the end, cells were collected using trypsin, washed and resuspended in PBS, followed by analyzation via Flow cytometry.

For analyzing the effect of BI-2865 on drug efflux in MDR cells, cells were pretreated by 10 µM Dox for 3 h, then discarded the medium and washed cells with PBS, added fresh medium with or without 8 µM BI-2865, incubated cells at 37 °C until cells were collected for detecting the residual DOX in cells through Flow cytometry at desired time points.

### P-gp ATPase assay

The activity of P-gp ATPase was evaluated via performing a colorimetric assay as described previously [31]. Crude membranes were separated from P-gp overexpressing High Five insect cells. The crude membrane protein (100 µg protein/mL) was incubated with varying doses of BI-2865 (0–5 µM) with or without 0.3 mM of sodium orthovanadate (Na_3_VO_4_) in the pH 6.8 buffer (composed of 50 mM KCl, 2 mM EDTA, 10 mM MgCl_2_, 1 mM DTT and 5 mM sodium azide) at 37 °C for 5 min. Then added Mg-ATP solution (5 mM) to initiate the ATP hydrolysis, and the reaction was kept for 20 min at 37 °C. Afterwards, 30 µL of 10% SDS solution was added to end the reaction. Followed by an addition of detection reagent (containing 10% ascorbic acid, 15 mM zinc acetate and 35 mM ammonium molybdate), incubating another 20 min at 37 °C, then measuring the absorbance of the mixture at 750 nm via the 96-well Fisher Scientific Multiskan FC Microplate Reader (Pittsburgh, PA, USA). The release of inorganic phosphate was quantified at the standard curve. The final BI-2865-stimulated P-gp ATPase activation was defined as the variation between the released amounts of inorganic phosphate from ATP in the presence and absence of Na_3_VO_4_.

### Photoaffinity labeling of P-gp with [^125^I]-iodoarylazidoprazosin (IAAP)

The photoaffinity labeling assay was conducted according to our previously established protocol [29]. Briefly, membrane protein was crudely extracted from the High Five insect cells expressing high P-gp, and taken 50 µg protein out for incubating with BI-2865 (0–5 µM) in the pH 7.5 Tris-HCl (50 mM) for 5 min at 25 °C. Under subdued light, added 3 nM [^125^I]-IAAP (2200 Ci/nmol) to the mixture for reacting another 5 min at 25 °C. Then radiolabeled samples with UV crosslinking on ice (365 nm). Afterwards, immunoprecipitated the radiolabeled P-gp with C219 antibody (Novus, Centennial, CO, USA). Finally, by using a Tris-acetate NuPAGE gel (7%), the samples were subjected to SDS-PAGE, dried and exposed overnight at -80 °C via Bio-Max MR film (Eastman Kodak Co., Rochester, NY). The IAAP labeling of P-gp captured radioactivity was assessed through the Storm 860 Phosphor Imager system (Molecular Dynamics, Sunnyvale, CA).

### Western blotting

Cells were harvested, and the proteins were extracted in RIPA buffer. Then quantified protein samples by using Pierce™ BCA Protein Assay Kit. Same amounts of proteins were separated via SDS-PAGE electrophoresis, followed by transferring to the 0.2 μm PVDF membranes. After blocking in the 5% nonfat milk for 1–2 h, probed the membranes by using desired primary antibodies and subsequently applicable secondary antibodies, with a final visualization by the ECL chemiluminescent detection.

### qRT-PCR

First, extracted the total RNA from cells by using the EZ-press RNA Purification Kit (B0004D). Reverse transcription assay was conducted by using the 4× Reverse Transcription Mix (EZB-RT2GQ). The obtained cDNA was used as templates in subsequent qRT-PCR assays by using the 2× SYBR Master Mix (A0001-R2). All steps were performed according to the corresponding instructions.

Primer sequences are presented here: P-gp (F: 5′-CAGGCTTGCTGTAATTACCCA-3′, R: 5′-TCAAAGAAACAACGGTTCGG-3′); GAPDH (F: 5′-GTCTCCTCTGACTTCAACAGCG-3′, R: 5′-ACCACCCTGTTGCTGTAGCCAA-3′).

### Detection of P-gp on the cell surface

Cells were incubated with or without 8 µM BI-2865 for 48 h, followed by collection and washing with chilled PBS. Then resuspended cells in 30 µL PBS (containing 0.5% BSA), and probed it with the FITC-marked P-gp antibody (10 µL) at 4 °C (45 min). Finally, washed again and resuspended cells in 300 µL PBS for Flow cytometry analysis.

### Immunofluorescence

KB, KBv200 cells (5 × 10^5^) and MCF7, MCF7/adr cells (3 × 10^5^) were planted to the glass bottom cell culture dishes. After attachment, cells were treated with 0 or 8 µM BI-2865 for 48 h, and then washed by PBS, fixed in 4% paraformaldehyde (PFA) for 20 min at room temperature (RT), additionally permeabilized in 0.1% Triton X-100 for 10 min. Cells were afterwards washed by using PBS again, followed by blocking with the 3% BSA at RT for 30 min and immunolabeling with the 1% P-gp antibody (22336-1-AP, Proteintech) overnight at 4 °C. Next, cells were in dark labeled with the 0.1% Alexa Fluor Plus 594-conjugated goat anti-rabbit IgG (A32740, Invitrogen) at RT for 1 h. Nuclei were stained by DAPI (1:1000) for 15 min. Finally, taken images by using Zeiss LSM 880 microscope with 63× oil lens.

### Molecular docking with P-gp

Molecular docking is an important method widely used to predict the interaction of small molecules with proteins. In this study, we docked BI-2865, Paclitaxel and Vincristine to the drug-binding area of P-gp to further analyze their interaction. Firstly, obtained the P-gp crystal structure from web of Protein Data Bank (https://www.rcsb.org/). Then, docking was completed via Auto Dock Vina software, and the visualization of results was achieved via the PyMOL 1.8.

### Accumulation of Paclitaxel in tumor analyzed by LC-MS

0.3 g tumor tissues were homogenized and extracted in 1500 µL methanol for 15 min at 70 Hz, then centrifuged at 12,000 rpm for 15 min to obtain the supernatants. Next, the supernatants were blown dry at room temperature, followed by the residues were dissolved again with 200 µL methanol, centrifuged again. Then, 2 µL of each sample’s final supernatant was injected into the high-resolution LC-MS (Synapt G2-Si, Waters) with PDA detector for further analysis.

A ACQUITY UPLC BEH C18 Column (1.7 μm, 2.1 × 100 mm) was used to separate the constituents in sample supernatant. The mobile phase (A) H2O with 0.1% formic acid, (B) acetonitrile were used in the following gradient elution profile: 0.00–1.00 min: 90% (A); 1.00–10.10 min: 10% (A); 10.10–12.00 min: 90% (A). Flow rate was maintained at 0.25 mL/min. Multiple reaction monitors (MRM) was used for quantification in both positive and negative modes.

### Statistical analysis

Every experiment in this study was independently repeated more than three times and data here represent the Mean ± SD. The significance between two data was statistically analyzed by Two-tailed Student’s t-test. *p < 0.05, **p < 0.01 and ***p < 0.001 mean statistically significant.

## Results

### BI-2865 enhanced the killing ability of anticancer drugs in P-gp overexpressing cells

**Fig. 1:**
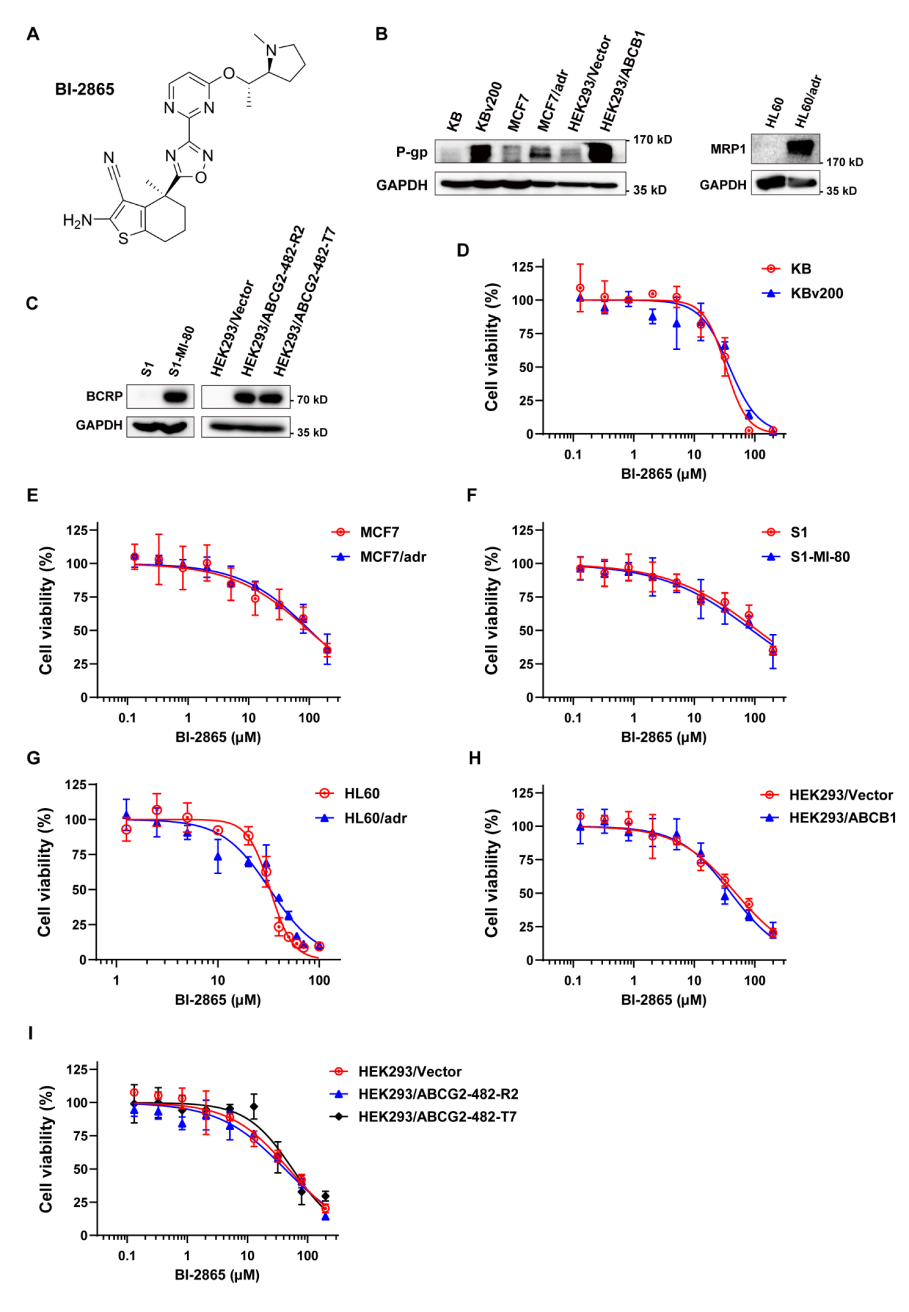
The structure and cytotoxicity of BI-2865. **A** Chemical structure of BI-2865. **B-C** Assessing the expression of P-gp, BCRP or MRP1 in the illustrated MDR cells and their parental sensitive cells by Western blotting with a loading control of GAPDH. **D**-**I** Indicated cell survival was measured by MTT assays after BI-2865 treating for 72 h. Data here represent Mean ± SD

The structure of BI-2865 was presented in Fig. 1A. Before the MDR reversing experiments proper start, Western blotting assays were firstly conducted to confirm the high P-gp expression in the resistant KBv200, MCF7/adr and HEK293/ABCB1 cells compared with their corresponding parental sensitive KB, MCF7 and HEK293/Vector cells (Fig. 1B). Moreover, other multidrug resistant cancer cells such as BCRP-overexpressing S1-MI-80 cells, HEK293/ABCG2-482-R2 cells and HEK293/ABCG2-482-T7 cells, MRP1-overexpressing HL60/adr cells and their corresponding parental cells were also confirmed (Fig. 1B-C) and used in the following experiments. For getting a suitable concentration of BI-2865 to manifest its sensibilization independent of cytotoxicity, MTT assays were done and as shown in Fig. 1D-I, concentrations of BI-2865 at 8 µM, 4 µM, 2 µM with at least 75% survived cells were chosen for subsequent reversal experiments.

We treated cancer cells with some traditional P-gp-, BCRP- or MRP1-substrate chemo-agents under different doses of non-significant cytotoxic BI-2865. By analyzing IC_50_ values of the indicated experimental groups in Table 1, MDR cells displayed more resistant than their sensitive cells, which is consistent with what previously reported [11]. What’s more, BI-2865 dose-dependently promoted the cytotoxic killing action of Paclitaxel, Vincristine (VCR), and Doxorubicin (DOX) in the drug-exposure-induced P-gp-overexpressing KBv200 and MCF7/adr cells, but did no effects in the corresponding parental sensitive cells, which was also repeated in the P-gp stably overexpressed HEK293/ABCB1 cells and its parental cells (Table 2). Meanwhile, the presence of BI-2865 neither turned out sensibilization to Mitoxantrone (MX) or Topotecan in the BCRP-overexpressing S1-MI-80, HEK293/ABCG2-482-R2 or HEK293/ABCG2-482-T7 cells, or sensibilization to Doxorubicin in MRP1-overexpressing HL60/adr cells, nor affected the IC_50_ of non-substrate control of Cisplatin.

**Table 1:** BI-2865 reversed MDR in P-gp overexpressing cancer cells

Compounds	IC_50_ ± SD (µM) (fold-reversal)			
	KB		KBv200 (P-gp)	
Vincristine	0.00204 ± 0.00026	(1.00)	0.16966 ± 0.01101	(1.00)
+ BI-2865 (2 µM)	0.00189 ± 0.00021	(1.08)	0.09284 ± 0.01950	(1.83) **
+ BI-2865 (4 µM)	0.00173 ± 0.00016	(1.18)	0.08494 ± 0.01467	(2.00) ***
+ BI-2865 (8 µM)	0.00207 ± 0.00030	(0.99)	0.05077 ± 0.01324	(3.34) ***
+ Verapamil (10 µM)	0.00181 ± 0.00024	(1.13)	0.00232 ± 0.00042	(73.05) ***
Paclitaxel	0.00677 ± 0.00116	(1.00)	0.09792 ± 0.01049	(1.00)
+ BI-2865 (2 µM)	0.00653 ± 0.00134	(1.04)	0.09409 ± 0.00750	(1.04)
+ BI-2865 (4 µM)	0.00641 ± 0.00035	(1.06)	0.10830 ± 0.02114	(0.90)
+ BI-2865 (8 µM)	0.00611 ± 0.00031	(1.11)	0.03508 ± 0.01114	(2.79) ***
+ Verapamil (10 µM)	0.00721 ± 0.00115	(0.94)	0.00658 ± 0.00069	(14.88) ***
Cisplatin	1.23642 ± 0.12800	(1.00)	1.57811 ± 0.06723	(1.00)
+ BI-2865 (8 µM)	1.13916 ± 0.11154	(1.09)	1.46765 ± 0.19305	(1.08)
	**MCF7**		**MCF7/adr (P-gp)**	
Doxorubicin	0.53289 ± 0.14013	(1.00)	5.30815 ± 1.44683	(1.00)
+ BI-2865 (2 µM)	0.45988 ± 0.05096	(1.16)	2.40757 ± 0.25817	(2.20) ***
+ BI-2865 (4 µM)	0.52120 ± 0.05554	(1.02)	1.76208 ± 0.10399	(3.01) ***
+ BI-2865 (8 µM)	0.58218 ± 0.10113	(0.92)	1.12453 ± 0.10592	(4.72) ***
+ Verapamil (10 µM)	0.43418 ± 0.02806	(1.23)	0.21585 ± 0.00338	(24.59) ***
Cisplatin	9.94431 ± 0.28265	(1.00)	11.82490 ± 0.68131	(1.00)
+ BI-2865 (8 µM)	8.84750 ± 1.13562	(1.12)	11.45492 ± 0.47511	(1.03)
	**S1**		**S1-MI-80 (BCRP)**	
Mitoxantrone	0.05962 ± 0.00540	(1.00)	17.98196 ± 1.40747	(1.00)
+ BI-2865 (2 µM)	0.06301 ± 0.01517	(0.95)	18.67101 ± 3.11650	(0.96)
+ BI-2865 (4 µM)	0.05123 ± 0.00414	(1.16)	17.03846 ± 1.20954	(1.06)
+ BI-2865 (8 µM)	0.05544 ± 0.00256	(1.08)	18.93954 ± 2.64097	(0.95)
+ Ko143 (30 nM)	0.06059 ± 0.00560	(0.98)	6.78781 ± 1.04839	(2.65) ***
Topotecan	1.54006 ± 0.14151	(1.00)	36.20099 ± 5.09340	(1.00)
+ BI-2865 (2 µM)	1.24845 ± 0.10042	(1.23)	39.99104 ± 1.13154	(0.91)
+ BI-2865 (4 µM)	1.62676 ± 0.29346	(0.95)	37.31575 ± 5.15159	(0.97)
+ BI-2865 (8 µM)	1.30874 ± 0.15460	(1.18)	34.20543 ± 6.54388	(1.06)
+ Ko143 (100 nM)	1.38275 ± 0.24371	(1.11)	17.94800 ± 2.01241	(2.02) **
Cisplatin	23.83792 ± 3.77089	(1.00)	35.45003 ± 1.25965	(1.00)
+ BI-2865 (8 µM)	25.04401 ± 3.89829	(0.95)	30.65739 ± 4.28209	(1.16)
	**HL60**		**HL60/adr (MRP1)**	
Doxorubicin	0.01169 ± 0.00187	(1.00)	2.83576 ± 0.20203	(1.00)
+ BI-2865 (2 µM)	0.01295 ± 0.00117	(0.90)	2.63314 ± 0.27518	(1.08)
+ BI-2865 (4 µM)	0.01142 ± 0.00114	(1.02)	2.40575 ± 0.19176	(1.18)
+ BI-2865 (8 µM)	0.01214 ± 0.00222	(0.96)	2.61154 ± 0.18295	(1.09)
+ MK571 (20 µM)	0.01203 ± 0.00058	(0.97)	0.96134 ± 0.20404	(2.95) ***
Cisplatin	0.46750 ± 0.04009	(1.00)	1.08095 ± 0.11996	(1.00)
+ BI-2865 (8 µM)	0.47758 ± 0.05297	(0.98)	1.14509 ± 0.19776	(0.94)

Cell survival was tested by MTT assays. Data represent the Means ± SD. (*p < 0.05, **p < 0.01 and ***p < 0.001, compared with the corresponding values obtained in the absence of inhibitor). The MDR reversal activity of BI-2865 in MDR cells was calculated by dividing the IC_50_ of cells treated only with anticancer drugs by that co-treated with drugs and BI-2865. Verapamil (a definite P-gp inhibitor), Ko143 (a definite BCRP inhibitor) or MK571 (a definite MRP1 inhibitor) was used as the positive control, and Cisplatin was as a non-substrate control

**Table 2:** BI-2865 reversed MDR in P-gp stable-overexpressing HEK293 cells

Compounds	IC_50_ ± SD (µM) (fold-reversal)			
	HEK293/Vector		HEK293/ABCB1	
Paclitaxel	0.04562 ± 0.00827	(1.00)	1.08259 ± 0.07259	(1.00)
+ BI-2865 (2 µM)	0.05385 ± 0.01028	(0.85)	1.16758 ± 0.12756	(0.93)
+ BI-2865 (4 µM)	0.04861 ± 0.00639	(0.94)	0.46256 ± 0.03636	(2.34) ***
+ BI-2865 (8 µM)	0.04607 ± 0.01240	(0.99)	0.29149 ± 0.04797	(3.71) ***
+ Verapamil (10 µM)	0.05103 ± 0.01968	(0.89)	0.09146 ± 0.00721	(11.84) ***
Doxorubicin	0.11912 ± 0.02515	(1.00)	1.27208 ± 0.17045	(1.00)
+ BI-2865 (2 µM)	0.13140 ± 0.01391	(0.91)	1.42503 ± 0.16530	(0.89)
+ BI-2865 (4 µM)	0.11940 ± 0.03121	(1.00)	0.96295 ± 0.10804	(1.32)
+ BI-2865 (8 µM)	0.10714 ± 0.01091	(1.11)	0.32889 ± 0.11350	(3.87) ***
+ Verapamil (10 µM)	0.13289 ± 0.02545	(0.90)	0.12869 ± 0.03399	(9.88) ***
Cisplatin	10.38305 ± 0.57421	(1.00)	18.39636 ± 2.70446	(1.00)
+ BI-2865 (8 µM)	10.30675 ± 2.46459	(1.01)	18.01007 ± 2.34893	(1.02)
	**HEK293/Vector**		**HEK293/ABCG2-482-R2**	
Mitoxantrone	0.24218 ± 0.01964	(1.00)	3.21068 ± 0.20074	(1.00)
+ BI-2865 (2 µM)	0.19500 ± 0.03006	(1.24)	3.46900 ± 0.98420	(0.93)
+ BI-2865 (4 µM)	0.22713 ± 0.02477	(1.07)	3.40070 ± 0.33370	(0.94)
+ BI-2865 (8 µM)	0.20461 ± 0.01405	(1.18)	3.42448 ± 0.30507	(0.94)
+ Ko143 (100 nM)	0.20760 ± 0.06184	(1.17)	0.67910 ± 0.09112	(4.73***)
	**HEK293/ABCG2-482-T7**			
Mitoxantrone	2.66021 ± 0.18924	(1.00)		
+ BI-2865 (2 µM)	2.43027 ± 0.47925	(1.09)		
+ BI-2865 (4 µM)	2.06656 ± 0.08574	(1.29)		
+ BI-2865 (8 µM)	2.48821 ± 0.51665	(1.07)		
+ Ko143 (100 nM)	0.97600 ± 0.06051	(2.73***)		

Cell survival was tested by MTT assays. Data represent the Means ± SD. (*p < 0.05, **p < 0.01 and ***p < 0.001, compared with the corresponding values obtained in the absence of inhibitor). The MDR reversal activity of BI-2865 in MDR cells was calculated by dividing the IC_50_ of cells treated only with anticancer drugs by that co-treated with drugs and BI-2865. Verapamil (a definite P-gp inhibitor) or Ko143 (a definite BCRP inhibitor) was used as the positive control, and Cisplatin was as a non-substrate control

These findings demonstrated that BI-2865 could significantly and selectively potentiate the cytotoxicity of traditional chemo-drugs on the P-gp-induced MDR cancer cells in vitro.

### BI-2865 improved the vivo efficacy of paclitaxel in P-gp overexpressing xenografts

**Fig. 2:**
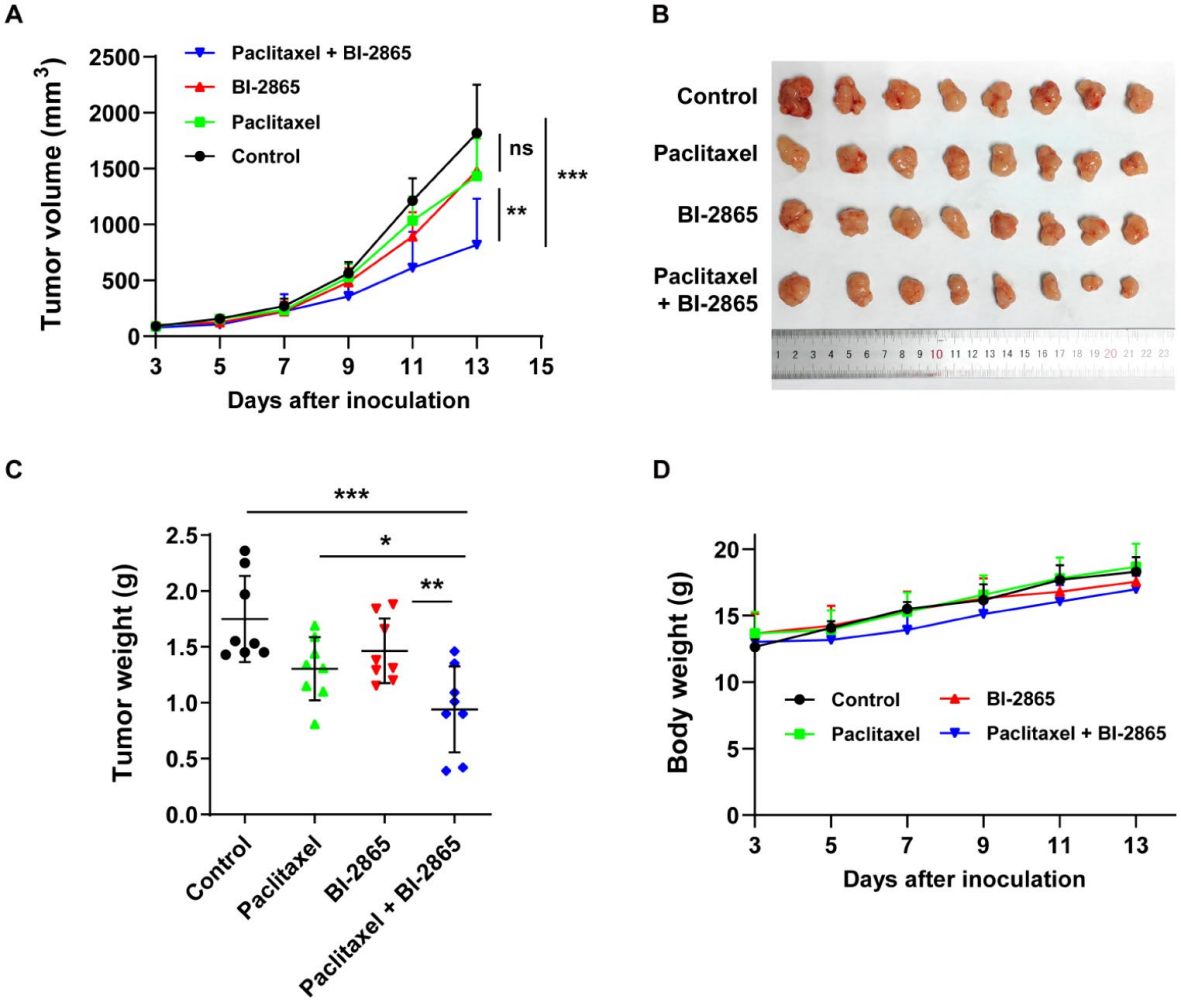
Enhanced paclitaxel anticancer activity by BI-2865 in MDR KBv200 xenograft model. **A**-**B** Alteration of tumor volume with time after KBv200 planted. **C** Tumor weight was evaluated at the end of experiments. **D** Changes of body weight with time after KBv200 inoculation. Data here represent Mean ± SD, and *p* < 0.05 means statistically significant (*p < 0.05, **p < 0.01 and ***p < 0.001, ns: no significant difference)

To further test the capability of BI-2865 on impairing MDR in vivo, the established P-gp overexpressing KBv200 cells were subcutaneously planted into the 3-week female BALB/C nude mice. When tumors grew near to 100 mm^3^ in volume, we grouped mice into four with randomization and gave indicated administration once every two days. As the result shown, there were no statistically markable differences in tumor growth analyzed in mice alone treated with saline, 15 mg/kg paclitaxel or 30 mg/kg BI-2865, while the co-administration of paclitaxel and BI-2865 significantly suppressed growth of tumor volume and weight without damaging mice body weight gain (Fig. 2A-D). Collectively, these results verified that BI-2865 could notably augment the therapeutic efficacy of paclitaxel in MDR xenografts mediated by P-gp overexpression in vivo.

### BI-2865 increased DOX accumulation in P-gp overexpressing MDR cells

**Fig. 3:**
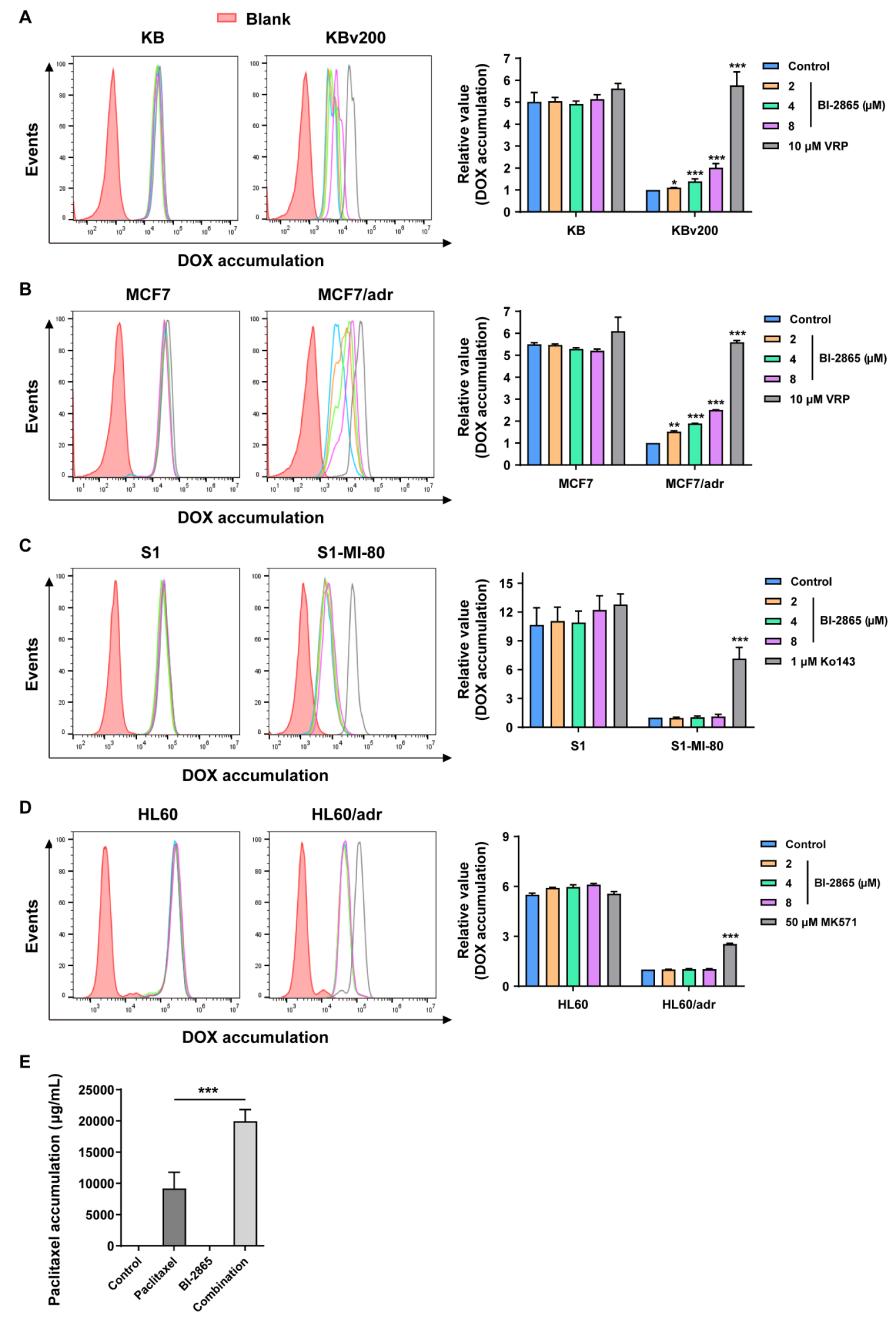
Effect of BI-2865 on accumulated DOX in cells or Paclitaxel in tumors. **A**-**D** Left: the DOX intracellular accumulation in P-gp induced MDR KBv200, MCF-7/adr cells, BCRP-induced MDR S1-MI-80 cells, MRP1-induced MDR HL60/adr cells and their parental sensitive cells, analyzing by Flow cytometry. Right: quantization for data in the left. The final value was normalized to the fluorescence intensity of MDR control cells. **E** The relative accumulation levels of Paclitaxel in KBv200 xenograft tumors treated by indicated drugs and their combination were evaluated by LC-MS (*n* = 6). Data here represent Mean ± SD, and *p* < 0.05 means statistically significant (*p < 0.05, **p < 0.01 and ***p < 0.001)

The results above indicated that BI-2865 could effectively sensitize high P-gp induced MDR cancer cells to the traditional chemotherapeutic agents. To further clarify the underlying mechanisms, we tested the DOX accumulating in MDR cells and their parental cells by Flow cytometry. Relative results were presented in Fig. 3A-B, similar to the positive control of Verapamil (VRP) (a definite inhibitor of P-gp), pretreatment of BI-2865 obviously elevated the residual DOX in KBv200 and MCF7/adr cells in a dose dependent manner, but didn’t affect that in the sensitive KB and MCF7 cells, which may attribute to the much more basal accumulation of DOX in the sensitive cells than in resistant cells. In addition, no apparent changes were observed in DOX accumulation of S1-MI-80 or HL60/adr cells pretreated by BI-2865 (Fig. 3C-D), which further suggested the selective reversal of BI-2865 on P-gp mediated MDR. Moreover, analysis of the Paclitaxel accumulation leves in the KBv200 xenografts’ tumor by LC-MS, also indicated that combination of BI-2865 and Paclitaxel remarkably increased the remains of Paclitaxel in tumor (Fig. 3E). These results emphasized the specific reversal effect of BI-2865 on P-gp mediated MDR cancers was achieved by increasing the accumulation of chemotherapeutic drugs in cells.

### BI2865 blocked the Dox efflux by competitively interacting with substrate-binding sites of P-gp

**Fig. 4:**
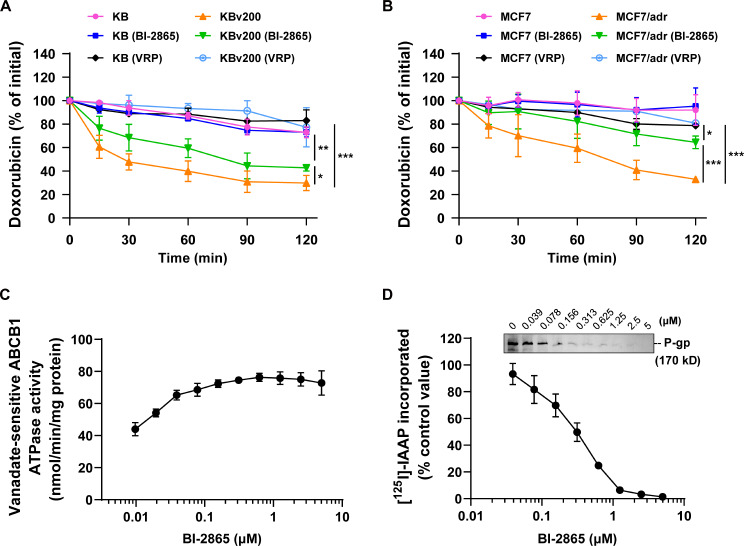
Effect of BI-3865 on DOX efflux, ATPase activity and [^125^I]-IAAP photoaffinity labeling of P-gp. **A**-**B** The residual intracellular DOX monitored by Flow cytometry at different time points indicated DOX efflux with time. **C** The vanadate-sensitive P-gp ATPase activity in the presence of the indicated dose of BI-2865. **D** BI-2865 competed for photolabeling of P-gp with [^125^I]-IAAP. Data here represent Mean ± SD

Given the ABC transporters mainly function to drive substrates out from cells, we assessed intracellular residual DOX at different time points after an initial 3 hours’ DOX (10 µM) incubation. As expected, the Flow cytometry tests showed that intracellular DOX decreased with time both in the sensitive KB cells and the resistant KBv200 cells, but DOX in KBv200 declined much faster than that in KB cells (Fig. 4A), which also validated the stronger efflux function of MDR cells mediated by P-gp overexpression. What’s more, the speed of DOX efflux in KBv200 got significantly slower after additional incubation with 8 µM BI-2865. In contrast, such an effect wasn’t observed in KB cells with BI-2865 incubation. The similar results were also obtained in efflux experiments by using MCF7 and MCF7/adr cells (Fig. 4B), which indicated that the markable effect of BI-2865 reversing MDR was achieved by restraining the P-gp substrate’s efflux and increasing its accumulation in cells.

Since the ABC transporting process is initiated by substrates binding to the drug-binding sites (TMDs) and driven by the energy molecules ATP binding to the nucleotide-binding domains (NBDs) of ABC proteins and hydrolysis, we next evaluated the vanadate-sensitive ATPase activity of P-gp at different doses of BI-2865. As shown in Fig. 4C, BI-2865-induced ATPase activity was dose-dependently increased, and remained about 75 nmol/min/mg protein when BI-2865 given more than 0.5 µM, suggesting a potential interaction of BI-2865 with the drug-binding sites of P-gp. Further evidence was given through assay of the P-gp photoaffinity labeling by [^125^I]-IAAP. The photo-affinity analogue of prazosin ([^125^I]-IAAP) is known to be transported by P-gp, and usually used to identify the competing binding of ABC substrates or inhibitors to P-gp transporters [29, 32]. In order to further confirm the binding of BI-2865 with P-gp, we extracted membrane protein containing P-gp and incubated that with [^125^I]-IAAP and desired BI-2865 of 0–5 µM, with the result showing that BI-2865 dose-dependently suppressed the binding of [^125^I]-IAAP with P-gp and the inhibition was reached 50% at about 0.3 µM BI-2865 (Fig. 4D). In general, these results claimed that BI-2865 might block the substrate drugs’ efflux by competing with drugs for the drug-binding sites of P-gp, leading to the increased ATP hydrolysis by P-gp ATPase, thus chemo drugs detained in cancer cells was increased.

### BI-2865 didn’t alter P-gp expression and its localization in MDR cells

**Fig. 5:**
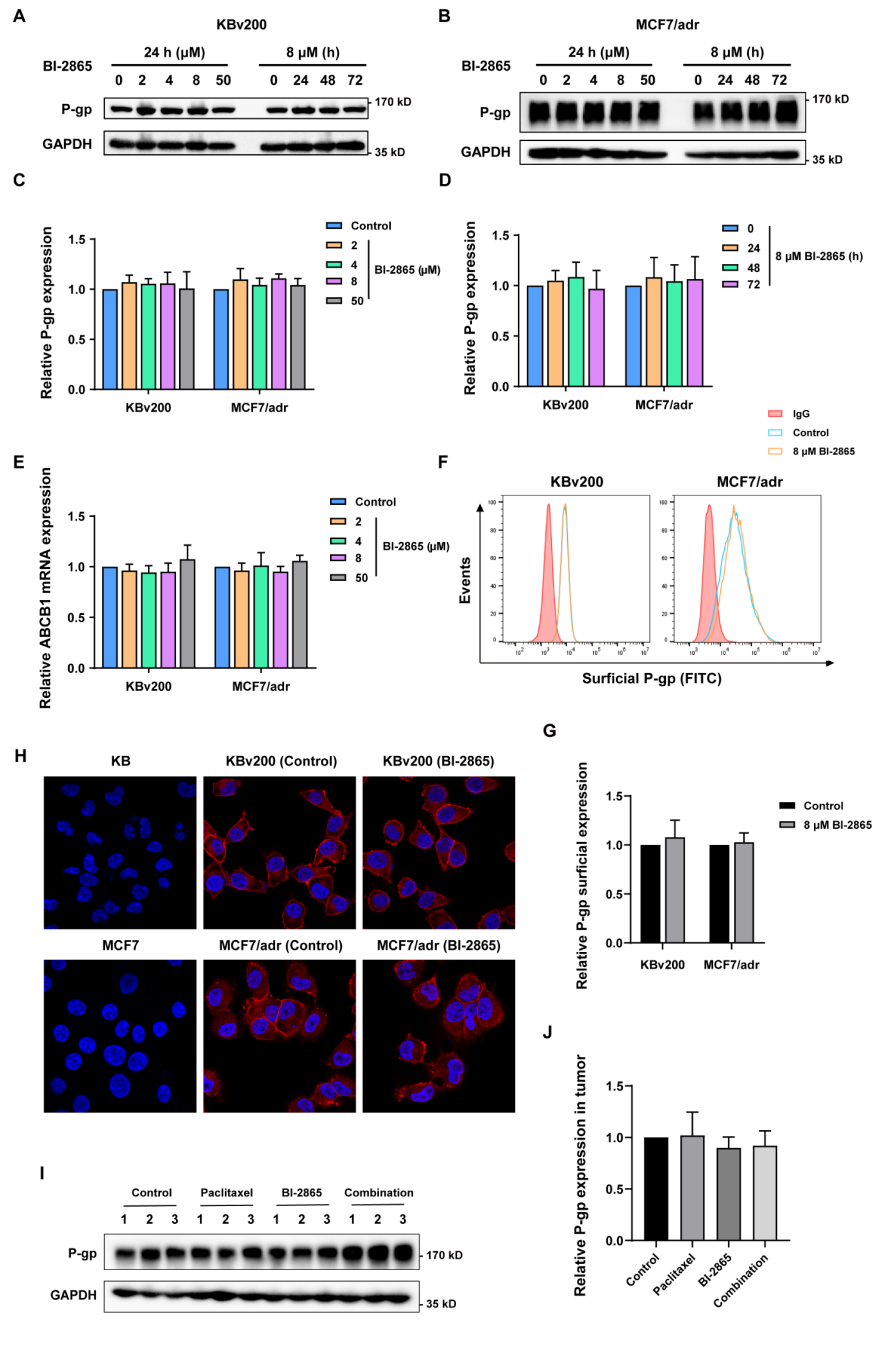
Effects of BI-2865 on P-gp expression and location in MDR cells. **A**-**B** Measuring the P-gp expression in MDR KBv200 and MCF7/adr cells with indicated treatment of BI-2865 by Western blotting. GAPDH was tested as a loading control. **C**-**D** Quantization of Fig. 5A-B. **E** ABCB1 mRNA expression was detected by qRT-PCR. **F** BI-2865 did not alter the P-gp amounts on cell surface analyzed via Flow cytometry. **G** Quantization for Fig. 5F. **H** Localization of P-gp was visualized by Laser Confocal Scanning Microscope with 63× oil immersion objective lens. (blue: DAPI stained nuclei, red: P-gp). **I** P-gp expression levels in KBv200 xenograft tumors treated by indicated drugs and their combination were evaluated by Western blotting. **J** Quantization for Fig. 5I. Data here represent Mean ± SD, and *p* < 0.05 means statistically significant (*p < 0.05, **p < 0.01 and ***p < 0.001)

Besides the activity of ABC transporters, their expression level in cancer cells is another more direct factor to regulate MDR, so Western blotting and qRT-PCR assays were carried out to analyze if BI-2865 made any changes in the expression of P-gp. Our data showed that incubation with BI-2865 did not influence P-gp expression both in protein and mRNA level, even increasing the concentration of BI-2865 to 50 µM or extending the incubating time to 72 h (Fig. 5A-E). The P-gp level on MDR cells’ surface was also evaluated via Flow cytometry, with a result showing non obvious variation caused by BI-2865 (Fig. 5F-G). In addition, the localization of P-gp in cells was visualized by immunofluorescence, turning out higher P-gp was expectedly observed on the membrane of MDR KBv200 and MCF7/adr cells compared with the sensitive KB and MCF7 cells, but P-gp on MDR cells’ membrane did hardly alter under BI-2865 treatment (Fig. 5H). What’s more, evaluating P-gp expression in the KBv200 xenografts’ tumor by Western blotting (Fig. 5I, J) also produced similar results. These data claimed that BI-2865 exerted its MDR reversal activity through directly blocking the efflux activity of P-gp, rather than modulating its expression and location.

### BI-2865 reversed MDR independent of suppressing the AKT and ERK pathways

**Fig. 6:**
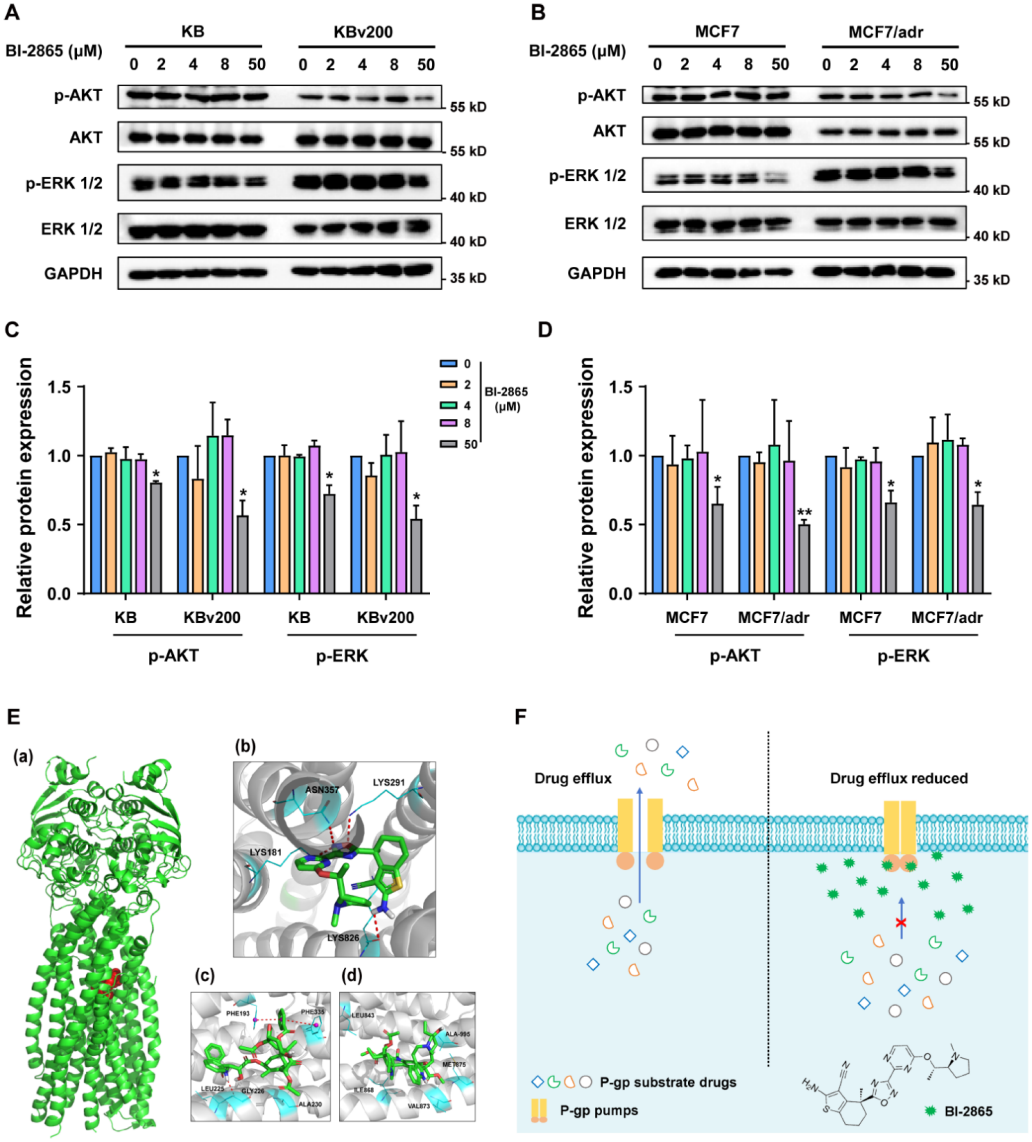
Effect of BI-2865 on AKT and ERK signaling, molecular docking and graphical abstract. **A**-**B** Analyzing the effect of BI-2865 on the activity of AKT and ERK signaling by Western blotting. GAPDH was tested as a loading control. **C**-**D** Quantization of Fig. 6A-B. Data represent Mean ± SD, and *p* < 0.05 means statistically significant (*p < 0.05, **p < 0.01 and ***p < 0.001). **E** The analysis of molecular docking. (Inset: a. overview of the binding of BI-2865 (red) with the TMD of P-gp (green). b-d. detailed view of BI-2865 (b), Paclitaxel (c) or Vincristine (d) docking to TMD of P-gp. Red dotted lines: interactions with P-gp.) **F** Graphical abstract: BI-2865 competitively interacted with the drug-binding domains (TMD) of P-gp to block the substrate drugs efflux and raise drugs’ amounts in cancer cells thus circumvent MDR

It is known that hyperactivation of KRAS stimulates the downstream AKT and ERK pathways, which were reported to attenuate resistance to chemo-agents in carcinomas [33], therefore, whether BI-2865 reversed MDR through activating AKT and ERK pathways was further identified here. Via experiments of Western blotting, BI-2865 was found having no effect on the phosphorylated AKT or ERK at its effective MDR reversal concentration of 2, 4 or 8 µM, but having inhibited the phosphorylated activation of the two pathways at a higher concentration of 50 µM (Fig. 6A-D). These results manifested that the reversal of P-gp mediated MDR by BI-2865 was independent of blocking AKT or ERK signaling.

### Molecular docking and binding site analysis

Considering BI-2865 might function via competing with the substrates to the drug-binding pockets of P-gp (Fig. 4C-D), the computer molecular docking with P-gp was performed to analyze the binding modes of BI-2865, P-gp’s substrates — Paclitaxel and Vincristine. The results were exhibited as Fig. 6E, in the most stable conformation of BI-2865 docking to the drug-binding domain of P-gp, BI-2865 occupied this area with the lowest energy of -9.2 kcal/mol, which is significantly lower than the binding energy of Paclitaxel to P-gp (-8.9 kcal/mol) and Vincristine to P-gp (-7.0 kcal/mol), indicating that P-gp might preferentially bind with BI-2865 for its higher affinity, which in turn hindered its binding with substrate drugs, thereby drugs’ efflux was limited and the chemotherapeutic efficacy was improved.

## Discussion

Multidrug resistance (MDR), developing in various carcinomas, protects cancer cells from cytotoxic killing by chemo-drugs and plays a crucial role in mattering the outcomes of cancer chemotherapy. Among the numerous complex factors resulting in MDR, the overexpression and enhanced efflux capability of ABC transporters exists in widely different drug-resistant cancer cells [34–36], even the abnormally high ABC transporters in carcinomas has been considered symbolic in the early development of chemo-resistance. ABC transporters are mainly in charge of the secretion and excretion of endogenous or exogenous substances from cells to maintain a normal physiologic homeostasis, but in tumor cells, the superabundant ABC transporters will recognize exogenous chemotherapeutic agents and pump them out to avoid therapeutic cytotoxicity, which then leads to resistance to many drugs and dismal prognosis in patients [37]. Therefore, attenuation of ABC transporters represents an attractive strategy to reduce MDR and advance efficacy of chemotherapy.

P-glycoprotein or P-gp was firstly identified in 1976 [38] and launched the subsequent wide study of various ABC transporters [7, 39]. To date, P-gp is still the most extensively researched ABC transporter about cancer MDR, whose overexpression has certainly been proved involved with the MDR in various cancers [1, 40]. P-gp modulators under clinical studies by far mainly fall into three generations; (a) The first-generation including verapamil, cyclosporine A, tamoxifen and quinidine, exhibits poor efficacy when combined with chemo-drugs, with narrow therapeutic window and life-threatening toxicity [35, 41]; (b) The second-generation including valspodar, emopamil, dexverapamil, gallopamil, cinchonine, biricodar, and Ro11-2933, being structurally analogous to the first-generation inhibitors, and exhibiting significant MDR reversal effect with lower off-target toxicity but causing accumulation of anticancer drugs in toxic amounts in organs for interacting with the co-administered anti-cancer drugs [42]; (c) The third-generation modulators was developed with high selectivity and efficacy, including tariquidar, laniquidar, elaquidar, zosuquidar and XR9051, but still unsuccessful in clinic for variabilities between patients [43, 44]. Development of the fourth-generation P-gp modulators are still ongoing, and there are still no relevant agents have been approved to sensitize P-gp mediated MDR cancers in clinic practice. Therefore, novel safe and effective efflux pump inhibitors are urgently needed.

KRAS is a kind of GTPase protein, firstly found on chromosome 12 of human lung cancer cells in 1982 [21]. Many documents clarified the mechanisms of KRAS driving cancers, in which KRAS keeps switching between the active GTP-bound form and the inactive GDP-bound form [45], the miscellaneous KRAS mutations widely existed maintains signaling constantly activated and stimulates cells’ proliferation, survival and metastasis then promotes the development of carcinomas [46]. However, after decades of hard work, the acknowledged crucial therapeutic target has been deemed challenging even undruggable for its smooth surface lacking suitable drug-pockets, until the surprising advances have made in the covalent KRAS G12C inhibitors, which specifically trap KRAS G12C bound GDP and restrain tumor growth in patients, for example, AMG510 (sotorasib) has been approved in clinical use. Nevertheless, non-G12C mutants constitute most KRAS mutations in cancer, which prompts efforts to develop agents targeting broad KRAS mutants. BI-2865 is a recently demonstrated novel pan-KRAS inhibitor significantly blocking a broad range of mutant KRAS with a promising development prospect [28]. Here, we firstly explored the ability of BI-2865 on overcoming MDR, expecting to discover its new applications and provide new sources for MDR modulators development.

Through MTT assays, we proved that BI-2865 could dose-dependently and selectively reverse the P-gp induced MDR in KBv200, MCF7/adr and HEK293/ABCB1 cells when combined with conventional chemotherapeutic agents, which was independent on its own cytotoxicity, while such a reversal effect wasn’t observed in the BCRP-induced MDR S1-MI-80 cells or MRP1-induced MDR HL60/adr cells treated by BI-2865 (Tables 1 and 2; Fig. 1). More importantly, the notable MDR reversal effects of BI-2865 has been further confirmed in KBv200 xenograft cancer model, and the vivo results also indicated a well-tolerance of BI-2865 and Paclitaxel co-administration (Fig. 2), suggesting low doses of BI-2865 could markedly sensitize P-gp mediated MDR tumors to chemo-drugs, and enhanced the therapeutic effect both in vitro and in vivo.

Studying the underlying mechanisms by Flow cytometry showed that BI-2865 treatment obviously inhibited the drug efflux and increased drug accumulation in MDR cells caused by P-gp not BCRP or MRP1 (Figs. 3 and 4A-B). In addition, through the assay of vanadate-sensitive P-gp ATPase, we found that BI-2865 stimulated ATPase activity in a dose dependent manner (Fig. 4C), meanwhile, the photoaffinity labeling assay showed that BI-2865 competed for the photo-affinity labeling of P-gp with [^125^I]-IAAP (Fig. 4D), indicating BI-2865 might be a competitive inhibitor of P-gp substrates.

Besides, the changes of P-gp expression and cellular location have also been assessed through Western blotting, qRT-PCR, Flow cytometry and immunofluorescence assays. Our results illustrated that no appreciable influence was made in the P-gp expression and cellular location by BI-2865 at its effective MDR reversal concentrations (Fig. 5). Moreover, the activity of KRAS downstream AKT and ERK signaling was tested through Western blotting, it turned out that circumvention of P-gp mediated MDR by BI-2865 was independent on its blocking of AKT or ERK signaling (Fig. 6A-D). Molecular docking also elucidated the potential preferential binding of P-gp TMD with BI-2865 than substrates as Paclitaxel and Vincristine (Fig. 6E), which verified the results of Fig. 4C-D that BI-2865 competitively inhibiting the substrate drugs binding to P-gp from the other perspective.

## Conclusions

Collectively, we discovered a new application of the novel pan-KRAS inhibitor — BI-2865 in reversing MDR in cancers, and systematically confirmed that BI-2865 significantly, safely and selectively enhanced the chemotherapeutic efficacy in the P-gp induced MDR tumors both in vitro and in vivo. Mechanistically, we revealed BI-2865, as a competitive inhibitor of P-gp substrates, might bind to the TMD of P-gp to limit the chemo-drugs efflux, thus increased the intracellular concentration of chemo-drugs, which did sensitize the MDR tumors (Fig. 6F).

This study hinted that BI-2865 could be used for combining with traditional chemo agents to improve therapeutic efficacy in clinical MDR cancers, and findings here would provide firm sustentation for further developing BI-2865 or its structural derivates as candidates to overcome MDR in cancer therapeutics.

### Electronic supplementary material

Below is the link to the electronic supplementary material.

Supplementary Material 1

## Data Availability

The data used in the present study are available from the corresponding authors upon reasonable request.

## Abbreviations

MDR: Multidrug resistance
NBD: Nucleotide-binding domain
TMD: transmembrane domain
ABC: ATP-binding cassette proteins
ABCB1: ABC subfamily member B1
P-gp: P-glycoprotein
MRP1: Multidrug resistance associated protein 1
ABCG2/BCRP: ABC subfamily member G2, or breast cancer resistance protein
VRP: Verapamil
VCR: Vincristine
DOX: Doxorubicin
MX: Mitoxantrone
[^125^I]-IAAP: Photo-affinity analogue of prazosin
PFA: Paraformaldehyde
RT: Room temperature
