# Supplementary material for: BI-2865, a pan-KRAS inhibitor, reverses the P-glycoprotein induced multidrug resistance in vitro and in vivo
Source: Cell Commun Signal. 2024 Jun 13;22:325. doi: 10.1186/s12964-024-01698-4 (PMC11170860; doi:10.1186/s12964-024-01698-4)
Supplement: Supplementary file 1 — Supplementary Material 1 [file 12964_2024_1698_MOESM1_ESM.pdf]

BI-2865, a pan-KRAS inhibitor, reverses the P-glycoprotein induced multidrug resistance in vitro and in vivo

Supplementary material（Original Western Blots）

Qihong Yang [1, 2], Kenneth Kin Wah To [3], Guilin Hu [4], Kai Fu [2], Chuan Yang [2], Shuangli Zhu [2], Can Pan [2], Fang Wang [2], Kewang Luo [1, *], Liwu Fu [2, *].

1. People's Hospital of Longhua, Shenzhen 518109, China.

2. State Key Laboratory of Oncology in South China, Guangdong Provincial Clinical Research Center for Cancer, Sun Yat-sen University Cancer Center, Guangzhou 510060, P. R. China.

3. School of Pharmacy, The Chinese University of Hong Kong, Hong Kong, 999077, China.

4. State Key Laboratory of Phytochemistry and Plant Resources in West China, Kunming Institute of Botany, Chinese Academy of Sciences, Kunming, 650201, China.

* Corresponding authors. Tel.: +8620-87343163; Fax: +862087343170;

E-mail addresses: kewangluo@126.com; fulw@mail.sysu.edu.cn.

Figure 1.

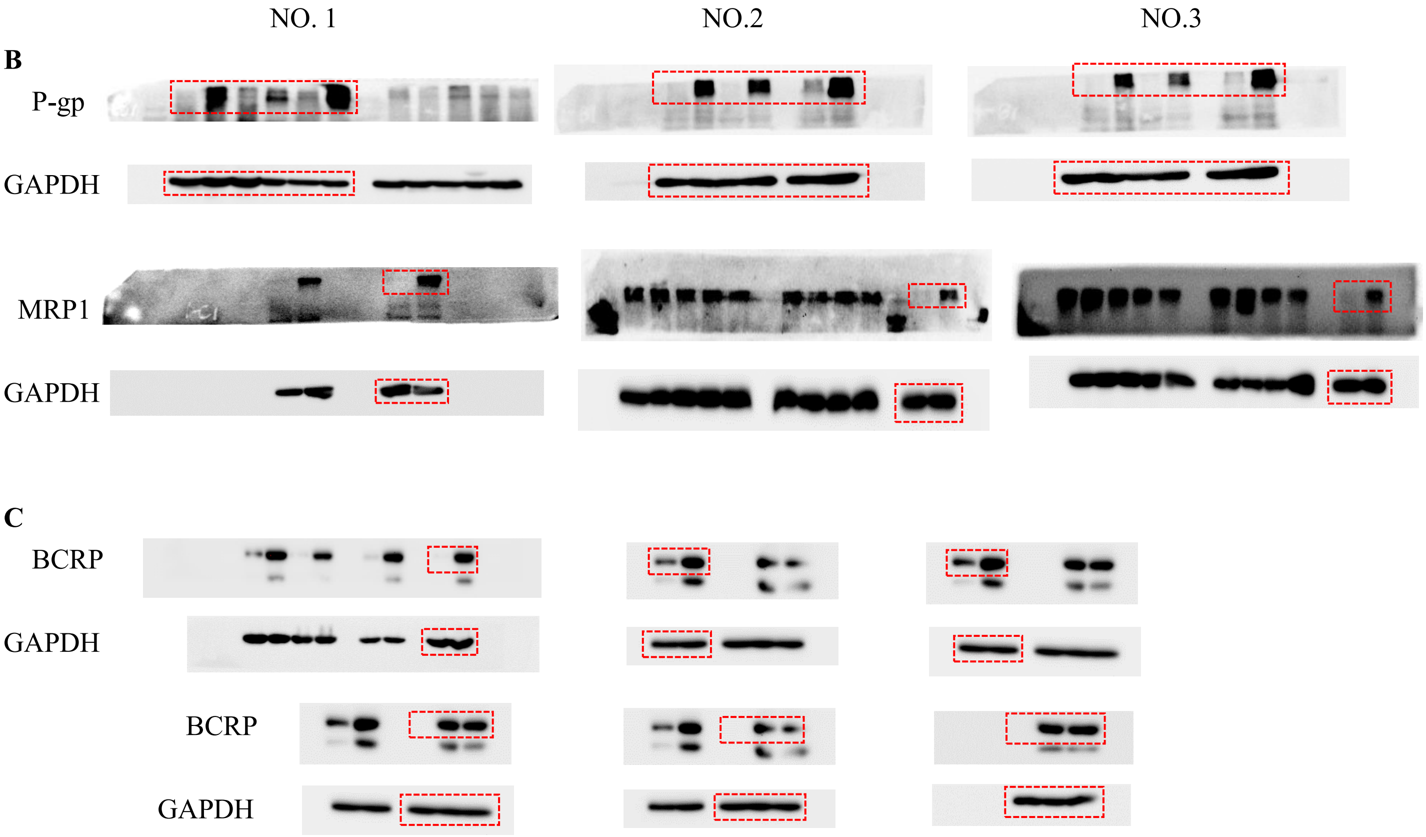

Figure 4.

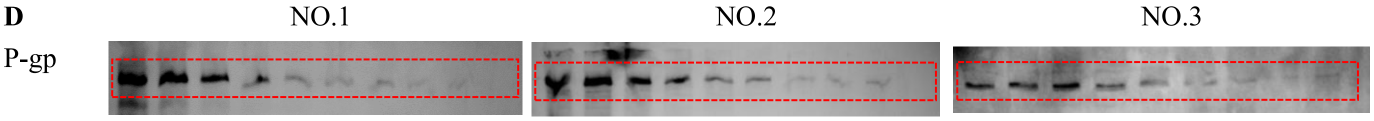

Figure 5.

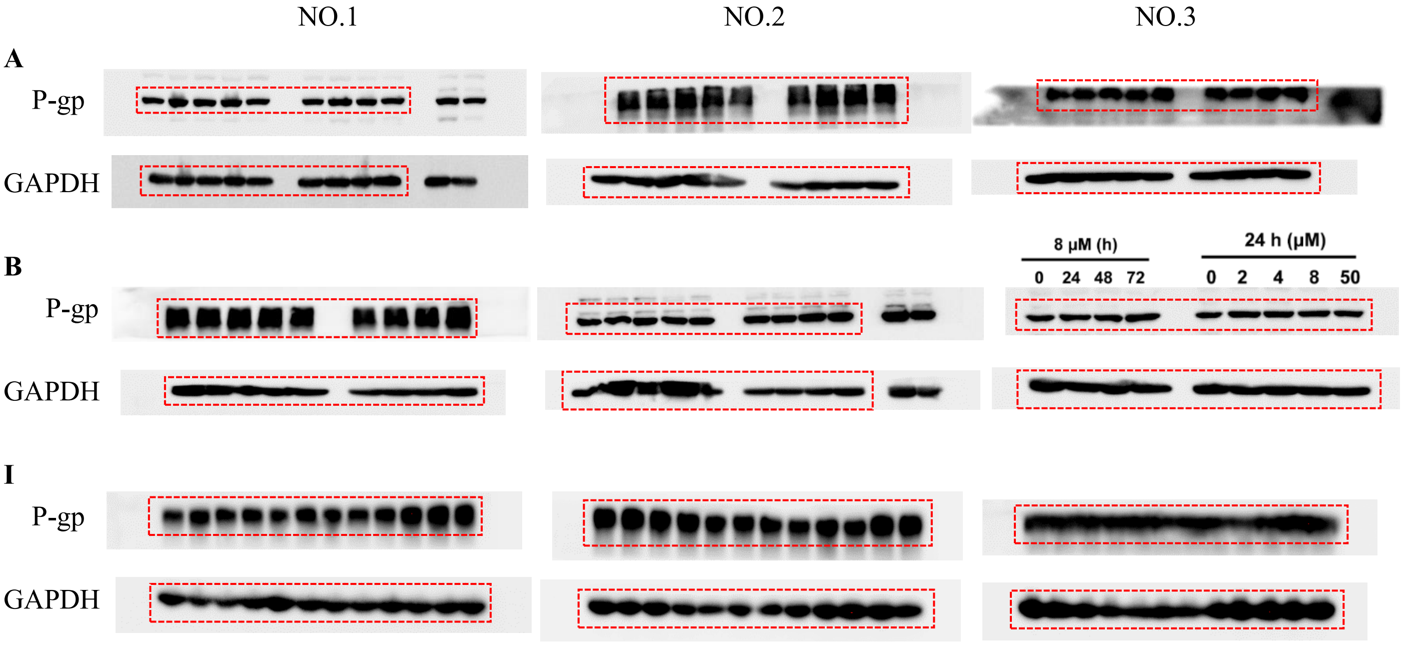

Figure 6.

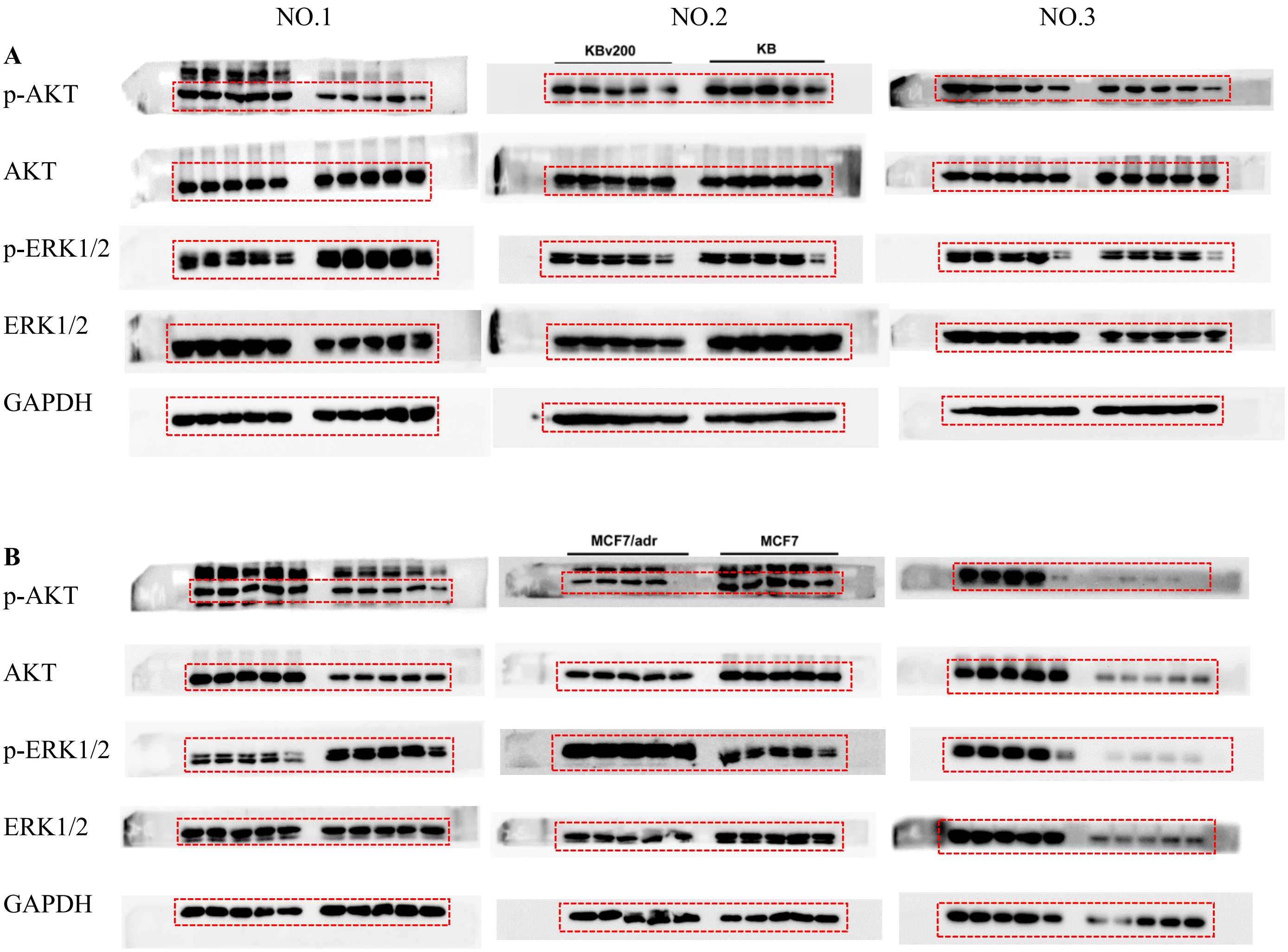

NO.1
NO.2
NO.3
A
p-AKT
KBv200
KB
AKT
p-ERK1/2
ERK1/2
GAPDH
B
p-AKT
MCF7/adr
MCF7
AKT
p-ERK1/2
ERK1/2
GAPDH
